# DNA Repair Deficient Chinese Hamster Ovary Cells Exhibiting Differential Sensitivity to Charged Particle Radiation under Aerobic and Hypoxic Conditions

**DOI:** 10.3390/ijms19082228

**Published:** 2018-07-30

**Authors:** Ian M. Cartwright, Cathy Su, Jeremy S. Haskins, Victoria A. Salinas, Shigeaki Sunada, Hao Yu, Mitsuru Uesaka, Hirokazu Hirakawa, David J. Chen, Akira Fujimori, Takamitsu A. Kato

**Affiliations:** 1 Department of Environmental & Radiological Health Sciences, Colorado State University, Fort Collins, CO 80523, USA; Ian.cartwright@ucdenver.edu (I.M.C.); Cathy.su@gmail.com (C.S.); haskins@rams.colostate.edu (J.S.H.); victoria_salinas@comcast.net (V.A.S.); 2 Mucosal Inflammation Program and Division of Gastroenterology, University of Colorado, Anshutz Medical Campus, Aurora, CO 80045, USA; 3 School of Engineering, The University of Tokyo, Tokyo 113-8656, Japan; sunada.mgen@mri.tmd.ac.jp (S.S.); yu@nuclear.jp (H.Y.); uesaka@nuclear.jp (M.U.); 4 Department of Molecular Genetics, Medical Research Institute, Tokyo Medical and Dental University, Tokyo 113-8510, Japan; 5 Department of Basic Medical Sciences for Radiation Damages, National Institute of Radiological Sciences, Chiba 263-8555, Japan; Hirakawa.Hirokazu@qst.go.jp (H.H.); Fujimori.akira@qst.go.jp (A.F.); 6 Department of Radiation Oncology, University of Texas Southwestern Medical Center, Dallas, TX 75390, USA; david.chen@utsouthwestern.edu

**Keywords:** ionizing radiation, DNA repair, LET, OER, RBE

## Abstract

It has been well established that hypoxia significantly increases both cellular and tumor resistance to ionizing radiation. Hypoxia associated radiation resistance has been known for some time but there has been limited success in sensitizing cells to radiation under hypoxic conditions. These studies show that, when irradiated with low linear energy transfer (LET) gamma-rays, poly (ADP-ribose), polymerase (PARP), Fanconi Anemia (FANC), and mutant Chinese Hamster Ovary (CHO) cells respond similarly to the non-homologous end joining (NHEJ) and the homologous recombination (HR) repair mutant CHO cells. Comparable results were observed in cells exposed to 13 keV/μm carbon ions. However, when irradiated with higher LET spread out Bragg peak (SOBP) carbon ions, we observed a decrease in the oxygen enhancement ratio (OER) in all the DNA of repair mutant cell lines. Interestingly, PARP mutant cells were observed as having the largest decrease in OER. Finally, these studies show a significant increase in the relative biological effectiveness (RBE) of high LET SOBP carbon and iron ions in HR and PARP mutants. There was also an increase in the RBE of NHEJ mutants when irradiated to SOBP carbon and iron ions. However, this increase was lower than in other mutant cell lines. These findings indicate that high LET radiation produces unique types of DNA damage under hypoxic conditions and PARP and HR repair pathways play a role in repairing this damage.

## 1. Introduction

Radiation-induced DNA damage results in chromosome aberrations, mutation, transformation, and cell death [1]. Ionizing radiation produces a variety of DNA damage, including, but not limited to: DNA double strand breaks; single strand breaks; base damages; and, crosslinks [2]. Due to the destructive nature of these DNA lesions, these cells have developed specific repair pathways to fix radiation induced DNA damage. Double strand breaks are the most lethal form of DNA damage and are primarily repaired by the non-homologous end joining (NHEJ) and homologous recombination (HR) repair pathways. These repair pathways are cell cycle dependent, with NHEJ functioning in G1/S/G2 and HR only functioning in S/G2. The loss of these repair pathways results in hypersensitivity to ionizing radiation and other DNA damaging agents [3,4]. Unrepaired or improperly repaired damage results in the formation of chromosome aberrations. The formation of dicentrics, translocations, and interstitial deletions, also results in the formation of micronuclei [5]. It has been well established that micronuclei can be utilized as a marker of radiation damage and radiation sensitivity [6,7,8,9].

The biological effects of ionizing radiation are heavily dependent on the presence of oxygen. In fact, the main mechanism of how low linear energy transfer (LET) radiation induces damage is through the formation of radical oxygen species [10,11]. The absence of oxygen in irradiated matter dramatically decreases damaging effects of radiation. Oxygen molecules chemically fix the DNA lesions produced by ionizing radiation. The degree of sensitization associated with oxygen is known as the oxygen enhancement ratio (OER). Typically, OER values are similar between all types of low LET radiation when using cell survival as an endpoint [12,13,14,15]. Additionally, it has been reported that OER is dependent on both LET and the presence or absence of DNA repair pathways [16]. As the LET of the radiation increases, OER values typically decrease [17,18]. High LET radiation such as alpha particles have been shown to have OER values of almost 1. This indicates that oxygen has almost no effect on cellular sensitivity to radiation [19].

High LET radiation, such as accelerated carbon–ions and the high-energy nuclei component of galactic cosmic rays, induce more biological effects, per absorbed dose, when compared to low LET radiation. High LET radiation is densely ionizing, which results in complex DNA damages that is not only difficult to repair, but may also require multiple DNA repair pathways to repair. As a result, high LET radiation has a higher RBE (Relative Biological Effectiveness) than low LET radiation. The loss of the NHEJ repair pathway results in high LET having a similar RBE as low LET radiation [20,21]. This suggests that the NHEJ repair pathway contributes to the repair of both low and high LET radiation damage. High LET radiation may produce DNA damage that oxygen reacts to differently and can potentially require different DNA repair pathways when the cell is under hypoxic conditions.

This study aims to investigate the role of various DNA repair pathways in response to DNA damage produced by high LET radiation under hypoxic condition. To do this, Chinese hamster ovary (CHO) cell lines with DNA repair defects in seven genes and four different radiation qualities were selected; cell survival was analyzed following their exposure to various LET radiation under aerobic and hypoxic conditions.

## 2. Results

### 2.1. Cell Survival

**Figure 1:**
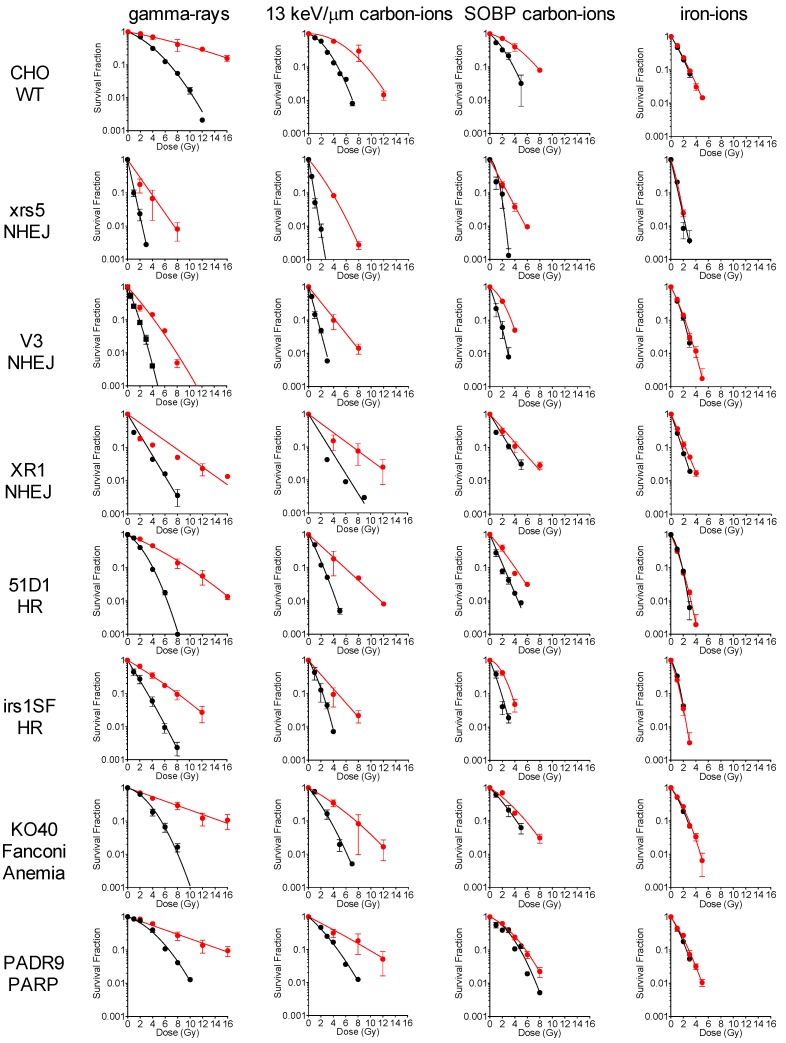
Cell survival curves generated for gamma-rays, carbon ion, carbon ion SOBP (Spread Out Bragg Peak), and iron ions irradiation under aerobic and hypoxic conditions. Black circles indicate the aerobic condition and red circles indicate the hypoxic condition. Error bars represent the standard error of the means. At least three independent experiments were carried out.

To determine the effects of hypoxia on the radiosensitivity of wild type and various DNA repair deficient cells, a variety of cells were irradiated with varying qualities of radiation under aerobic and hypoxic conditions. When exposed to low LET gamma-rays, there was a significant decrease in the radiation sensitivity of all cell lines under hypoxic conditions (Figure 1). It was observed that gamma-rays and low LET 13 keV/µm carbon ions showed a similar loss of radiation sensitivity under hypoxic conditions (Figure 1). When exposed to higher LET SOBP (Spread Out Bragg Peak) carbon ion radiation, a larger decrease in hypoxia associated radiation resistance in the HR and PARP (poly(ADP-ribose) polymerase) mutants was observed—as compared to the NHEJ and WT (wild type) cell lines (Figure 1). Finally, when cells were irradiated with high LET iron ions, the hypoxia associated radiation resistance was lost in all cell lines (Figure 1). Regression curves were determined for each cell line and a survival fraction of 2 Gy (SF2) was calculated for each radiation condition (Table 1). SF2 data further supported the conclusions drawn from the survival curves in Figure 1.

**Figure 2:**
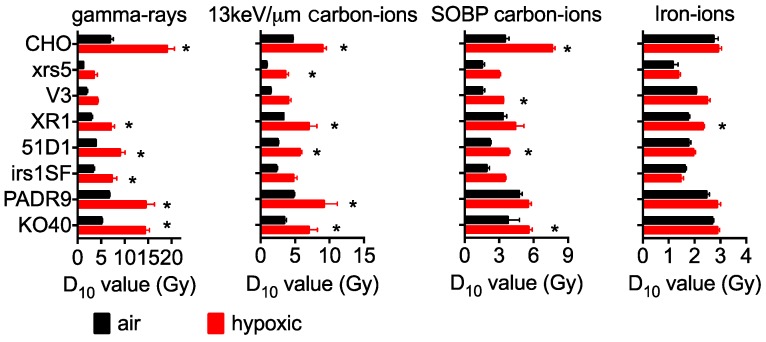
D_10_ values calculated from survival curves in different qualities of radiation. D_10_ values are the mean ± standard error of the means. * Indicates statistically significant differences between aerobic and hypoxic irradiation conditions (*p* < 0.05).

Additionally, linear quadratic regression was utilized to calculate the D_10_ values for wild type and DNA repair deficient cell lines (Figure 2). As shown previously, NHEJ mutants were the most radiation sensitive cells, followed by HR mutants, PARP, and FANCG (Fanconi Anemia complementation group G) mutants, with the CHO of wild type being the most radioresistant [22]. Hypoxic conditions resulted in an increased radioresistance when exposed to gamma-rays, carbon ion LET 13 keV/μm, and carbon ion SOBP—not, however, for iron ions.

### 2.2. RBE in Aerobic Condition

**Figure 3:**
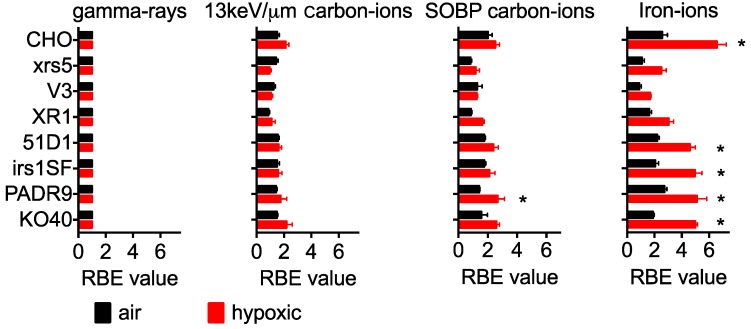
RBE values calculated from survival curves in different qualities of radiation. RBE values are the mean ± standard error of the means. * Indicates statistically significant differences between aerobic and hypoxic irradiation conditions (*p* < 0.05).

RBE values were separately calculated for both aerobic and hypoxic conditions. Under aerobic conditions, the RBE values for CHO wild type cells increased with the increasing LET; the max RBE observed was 2.5 when exposed to iron ions (Figure 3). The three NHEJ repair deficient cells showed almost no change in RBE as the LET increased. RBE values were approximately 1, except for XR1 cells, which had an RBE of 1.85 for iron ions. HR deficient cells showed a slight increase in RBE, but not to the same extent as the wild type cells. When exposed to iron ions, the RBE values were observed at 2.20 and 1.98 for 51D1 and irs1SF, respectively. KO40 cells showed a similar trend to the HR repair deficient cells. PADR9, however, showed a similar trend to the wild type cells. PADR9 had an RBE value of 2.66 when irradiated to iron ions, which was slightly higher than in wild type cells.

### 2.3. RBE for Hypoxic Condition

RBE values obtained under hypoxic conditions displayed some interesting trends (Figure 3). As the LET increased, the RBE values increased more significantly under hypoxic conditions than under aerobic conditions. Wild type cells had an RBE value of over 2 when irradiated with 13 keV/μm carbon ions and further increased to 6.52 when irradiated with iron ions. While the NHEJ deficient cells showed almost no increased RBE value under aerobic conditions, increased RBE values were observed when irradiated with SOBP carbon ions and iron ions. xrs5, V3, and XR1 had observed RBE values of 2.70, 1.89, and 3.32 for iron ion irradiation, respectively. HR deficient cells, KO40, and PADR9 cells showed similar changes in RBE to wild type cells. Hypoxic RBE values were greater than 2 for SOBP carbon ions and 5 for iron ions for all cell lines. These values were statistically significant when compared to the air RBE values of SOBP carbon ion exposed cells.

### 2.4. OER

**Figure 4:**
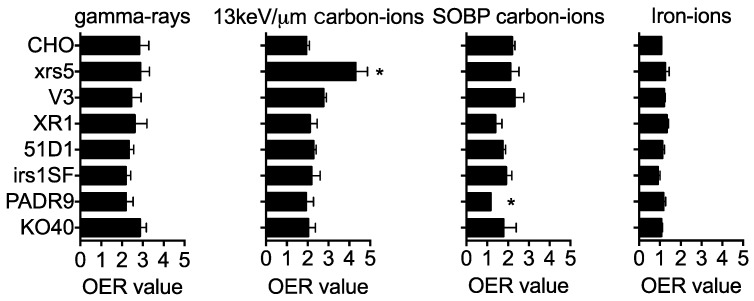
OER values calculated from survival curves in different qualities of radiation. RBE values are the mean ± standard error of the means. * Indicates statistically significant differences from wild type data (*p* < 0.05).

To determine the effect of oxygen on radiation sensitivity, OER values were calculated from the D_10_ values. When exposed to gamma-rays, hypoxic conditions resulted in the radiation resistance of both the wild type and DNA repair deficient cell lines (Figure 4). OER values for wild type cells decreased as the LET increased, ranging from 2.83 with gamma radiation to 1 for iron–ions. Two of the NHEJ repair deficient cells (V3 and XR1) showed similar patterns to the wild type cells; whereas, xrs5 cells showed a statistically significant difference in OER value when compared to wild type cells exposed to 13 keV/μm carbon ions. The HR, PARP, and FANCG mutants showed a similar trend to the wild type cells when irradiated with low LET radiation. The main difference arose when cells were irradiated with SOBP carbon ions. Several of the DNA repair mutants had lower OER values than wild type cells when irradiated with SOBP carbon ions. The largest difference was observed in HR and PARP mutants. PARP deficient mutants showed the most statistically significant difference when compared to the wild type controls. These cell-line specific differences were not observed when cells were irradiated with 200 keV/μm iron ions.

### 2.5. Micronuclei Formation

**Figure 5:**
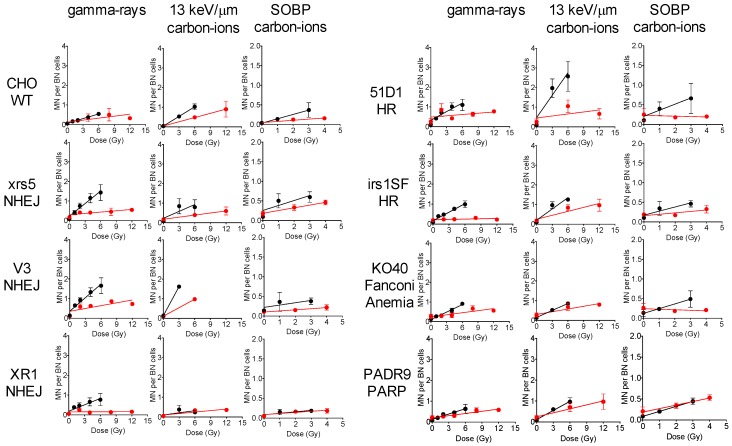
Micronuclei formation assay after different qualities of irradiation in the presence or absence of oxygen. Black circles indicate aerobic condition and the red circles indicate hypoxic condition. All experiments were carried out three times independently.

**Figure 6:**
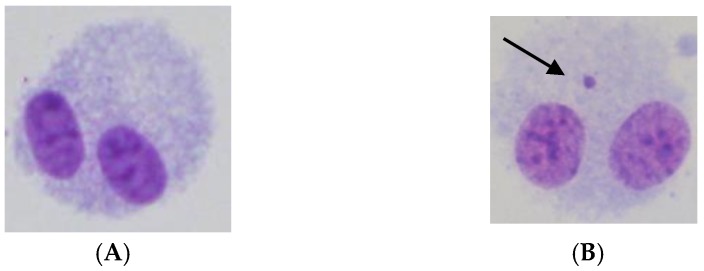
Representative images of micronuclei formation. (**A**) Unirradiated binucleated CHO cell without micronuclei. (**B**) 6 Gy of gamma-ray irradiated binucleated CHO cell with a micronucleus. The arrow indicates the micronucleus.

To further investigate the effects of oxygen on radiation sensitivity, we analyzed the formation of micronuclei in wild type and DNA repair mutants irradiated with gamma-rays, 13 keV/μm carbon ions, and SOBP carbon ions under both aerobic and hypoxic conditions (Figure 5 and Figure 6). When irradiated with gamma-rays under aerobic conditions, all cell lines had a dose-dependent increase in the observed micronuclei. DNA repair mutants had an increase in the number of micronuclei when compared to wild type cells. NHEJ mutants were observed to have had the highest number of micronuclei. All cell lines showed a statistically significant decrease in observed micronuclei when irradiated with gamma-rays under hypoxic conditions. Both the NHEJ and HR mutants showed the largest decrease in micronuclei formation when exposed to hypoxic conditions. Similar trends were observed when cells were irradiated with 13 keV/μm carbon ions, except for XR1, KO40 and PADR9 cells. These cells showed smaller differences in the number of micronuclei formed under aerobic and hypoxic irradiation conditions, as compared to the wild type cells. When irradiated with SOBP carbon ions, it was observed that XR1, KO40, and PADR9 cells did not experience a decrease in micronuclei formation under aerobic, as compared to, hypoxic conditions. This supports the lower OER values observed in Figure 4.

## 3. Discussion

Our findings suggest that the repair of high LET radiation-induced damage under hypoxic conditions requires not only the HR repair pathway, but also PARP. These findings are potentially of interest due to the hypoxic nature of tumors. One of the leading reasons for radiotherapy failure is tumor hypoxia [23]. It is quite common to find a portion of cells within a tumor to be either acutely or chronically hypoxic. One study showed that 70% of head and neck tumors and 63% of breast tumors were hypoxic and that tumor hypoxia resulted in high rates of radiotherapy failure [24]. Additionally, recent studies have shown that cancer stem cells colonize these hypoxic regions and have the potential to repopulate a tumor if it is not targeted with adequate radiation [25,26,27]. Traditionally, dose fractionation has been used to overcome this acute and chronic hypoxia via reoxygenation of the tumor core [28,29,30]. The issue with dose fractionation is tumor repopulation between fractionations [31,32,33]. In addition to dose fractionation, radiation sensitizers and high LET radiation has been utilized to overcome tumor hypoxia with limited success [34,35,36,37,38]. It has been reported that the inhibition of DNA repair pathways reduces the OER of gamma-rays, which we observed in Figure 4 [16].

We confirmed these findings, showing that the inhibition of all DNA repair pathways enhanced the effect of radiation under hypoxic conditions (Figure 1). Most notably, the inhibition of DNA-PKcs, XRCC4, and PARP showed the largest increase in radiation sensitivity under hypoxic conditions (Figure 1, Table 1). These findings indicate that the quality of radiation played a large role in OER. As the LET increased, we observed a decrease in hypoxia associated radiation resistance. It has been well established that, as LET increases, the complexity of radiation-induced damage increases [39]. Based on our data, we demonstrate a notable decrease in OER in HR and PARP mutant cell lines with adjuvant SOBP carbon ion irradiation and concurrent hypoxia. Higher LET radiation induces an increase in both the amount and type of DNA damage, which requires more than the NHEJ pathway to repair. It has been reported that PARP is required for a fully functional HR response [40,41,42,43]. These reports, taken in combination with our data, suggest that under hypoxic conditions, higher LET radiation produces complex DNA damage, which requires a functional HR pathway to fully repair. The reduction in OER was observed in all cell lines when LET reached its maximum biological effectiveness of above 100 keV/μm for iron ions. This observation supports the earlier finding that high LET iron ions do not rely on the presence of oxygen to cause DNA damage and cell death [44,45]. Despite the increased sensitivity of cells exposed to iron ions, this type of radiation is not clinically relevant. Of clinical significance is the findings observed in cells exposed to SOBP carbon ions, which are currently in use at 10 facilities [46]. It is interesting to note that mutations in XRCC4, HR, and PARP showed the highest sensitization under hypoxic conditions when irradiated with SOBP carbon ions. Specifically, PARP mutations had the highest RBE value and the lowest OER value in the cell lines tested. These findings indicate that PARP inhibition mitigates hypoxia associated radiation resistance. These findings in SOBP carbon ion therapy further supports the use of PARP inhibitors when combined with radiation therapy [47].

Our study suggests that high LET carbon–ion radiation therapy can be enhanced by adding an HR inhibitor, or specifically, a PARP inhibitor [48]. The addition of a PARP inhibitor sensitizes the entire tumor, including the hypoxic core. By overcoming the radioresistant hypoxic regions of a tumor via PARP inhibitors, this may introduce a potential reduction in overall fractionated dose, or conveniently no dosing at all. Given the added complexity, it is still unknown whether these findings would translate into an in vivo model. Additionally, further research into what role PARP plays in the repair of complex DNA damage caused by high LET radiation is needed. Whether PARP inhibitors are more effective when combined with high LET radiation than they are with low LET gamma or proton therapy also needs to be determined. It is worthy to mention that this investigation is limited to in vitro cell culture systems with two endpoints and also that in vivo studies should be conducted to determine the effectiveness of PARP inhibitors as a hypoxia radiation sensitizer in vivo.

## 4. Materials and Methods

### 4.1. Cell Culture

CHO wild type (CHO 10B2), and DNA repair deficient CHO mutant xrs5 (Ku80) [49], XR-1 (XRCC4) [50], and PADR9 (PARP) [51] were kindly supplied by Dr. Joel Bedford of Colorado State University (Fort Collins, CO, USA). DNA repair deficient CHO mutants, V3 (DNA-PKcs) [52], 51D1 (Rad51D) [53], irs1SF (XRCC3) [54], and KO40 (FANCG) [55] were kindly supplied by Dr. Larry Thompson at the Lawrence Livermore National Laboratory (Livermore, CA, USA). Cells were maintained in Alpha MEM (Hyclone, ThermoFisher, Waltham, MA, USA) with 10% heat inactivated Fetal Bovine Serum (Sigma, St. Louis, MO, USA), antibiotics (Anti-Anti; Invitrogen, Grand Island, NY, USA), and were cultured in 37 °C incubators with 5% CO_2_ and humidity.

Hypoxic conditions were maintained as previously published [56,57]. Hypoxia was achieved using the AnaeroPack system (Mitsubishi Gas Chemical, Tokyo, Japan) [58]. The cell cultures were placed into an airtight container with AnaeroPack oxygen absorbing and CO_2_ generating agents to reduce the O_2_ concentration to less than 1%. The cell cultures were treated in this hypoxic chamber for three hours at 37 °C before irradiation.

### 4.2. Irradiation

Gamma-ray irradiation was performed at Colorado State University with a J.L. Shepherd Model Mark I-68 nominal 6000 Ci ^137^Cs irradiator (J.L. Shepherd and Associates, San Fernando, CA, USA) at room temperature (20 °C) [59,60]. The dosage rate was 2.5 Gy/min for cell survival and micronuclei experiments. Particle-based irradiation experiments were carried out at the National Institute of Radiological Sciences (NIRS) in Chiba, Japan. Carbon ions and iron ions were accelerated to 290 and 500 MeV/n, respectively, using the Heavy Ion Medical Accelerator in Chiba (HIMAC) [60]. Specifics regarding the beam characteristics of the particle radiation, biological irradiation procedures, and dosimetry have been depicted previously [21,61,62]. Carbon ions were accelerated at 290 MeV/n of initial energy with 13 keV/μm on entrance or spread out with a ridge filter for 6 cm width of SOBP (spread out Bragg peak) [60]. The monolayer cell culture was irradiated at the center (50 keV/μm of average LET) within the SOBP at a distance of 119 mm from the entrance [57]. Monoenergetic 500 MeV/n iron ions which have a LET value of 200 keV/μm on entrance. Dose rates for carbon and iron ions irradiation were set at 1 Gy/min.

### 4.3. Cell Survival Colony Formation Assay and RBE, OER, and SF2 Calculation

After irradiation, cells were trypsinized and plated to form colonies. Colonies were fixed and stained 8 days later using 100% ethanol followed by 0.1% crystal violet. Macroscopic colonies containing more than 50 cells were marked as survivors [63]. Cell survival curves were drawn from cell survival fraction by Graphpad Prism 6 (GraphPad, La Jolla, CA, USA) with linear quadratic regression model. D_10_ values (radiation dose to achieve 10% cell survival) were obtained from regression curves. RBE and OER values were calculated from D_10_ values. Gamma-rays were used as a standard radiation for the RBE calculation. SF2 values were calculated from a regression model.

### 4.4. Micronuclei Formation Assay

Cells were irradiated and cultured in 4 μg/mL of Cytochalasin B (Sigma, St Louis, MO, USA) for 22 h [64]. Harvested cells were suspended in 5 mL of 75 mM KCl solution, centrifuged, and fixed in 3:1 methanol acetic acid solution and formaldehyde (ThermoFisher, Waltham, MA, USA). Cells were dropped onto slides and allowed to air dry at room temperature. Slides were stained in 5% Giemsa solution in GURR solution (Invitrogen, Grand Island, NY, USA) for 5 min. A total of 300 binucleated cells were scored per treatment dosage to obtain micronuclei per binucleated cells.

### 4.5. Statistics

All experiments were carried out at least two times and error bars indicate standard error of the means. Data was analyzed using Prism 6 software for one-way ANOVA analysis. *p*-values < 0.05 were categorized as significant differences.

## 5. Conclusions

A hallmark of hypoxia is radiation resistance. In this study we have shown that DNA repair deficient cells are more sensitive to high LET radiation under hypoxic conditions than in wild type controls. Interestingly, PARP deficient mutants showed similar OER values to HR mutants. These mutants had lower OER values than wild type controls when irradiated with carbon ion SOBP. Additionally, significantly higher RBE values were observed in HR, Fanconi Anemia, and PARP deficient cells with iron ion irradiation. NHEJ deficient cells also showed increased RBE values under hypoxic irradiation conditions. This study suggests that DNA repair inhibition may be a potential strategy for increasing the effectiveness of carbon ion radiotherapy when targeting the hypoxic regions of a tumor.

## Figures and Tables

**Table 1:** SF2, survival fraction at 2 Gy, for different qualities of radiation.

		CHO	xrs5	V3	XR1	51D1	irs1SF	PADR9	KO40
gamma-rays	aerobic	0.631	0.020	0.092	0.238	0.426	0.250	0.657	0.618
	hypoxic	0.840	0.291	0.362	0.543	0.676	0.595	0.737	0.735
carbon-ions 13 keV/μm	aerobic	0.538	0.006	0.041	0.250	0.154	0.147	0.423	0.302
	hypoxic	0.898	0.322	0.340	0.527	0.452	0.375	0.638	0.603
carbon-ions SOBP	aerobic	0.371	0.060	0.054	0.241	0.131	0.081	0.551	0.372
	hypoxic	0.710	0.201	0.369	0.379	0.339	0.428	0.583	0.520
iron-ions 200 keV/μm	aerobic	0.201	0.021	0.110	0.070	0.074	0.042	0.174	0.204
	hypoxic	0.222	0.025	0.138	0.131	0.081	0.037	0.223	0.248

## Abbreviations

CHO	Chinese Hamster Ovary
D_10_	Dose to achieve 10% cell survival
SF2	Survival Fraction at 2 Gy
LET	Linear Energy Transfer
RBE	Relative Biological Effectiveness
OER	Oxygen Enhancement Ratio
NIRS	National Institute of Radiological Sciences
HIMAC	Heavy Ion Medical Accelerator in Chiba
SOBP	Spread Out Bragg peak
ANOVA	Analysis of valiance
PARP	Poly (ADP-ribose) polymerase
FANC	Fanconi Anemia
NHEJ	Non Homologous End Joining
HR	Homologous Recombination
